# Diagnosis and treatment patterns among patients with newly diagnosed *Helicobacter pylori* infection in the United States 2016–2019

**DOI:** 10.1038/s41598-023-28200-3

**Published:** 2023-01-25

**Authors:** Shailja Shah, Katherine Cappell, Robert Sedgley, Corey Pelletier, Rinu Jacob, Machaon Bonafede, Rena Yadlapati

**Affiliations:** 1 University of California, San Diego, CA USA; 2 VA San Diego Healthcare System, San Diego, CA USA; 3 Veradigm, Chicago, IL USA; 4 Phathom Pharmaceuticals, Florham Park, NJ USA

**Keywords:** Stomach diseases, Antimicrobial therapy, Helicobacter pylori, Outcomes research

## Abstract

Approximately 36% of the United States (US) population is infected with *Helicobacter pylori* (HP), a known major risk factor for peptic ulcer disease and gastric cancer. HP eradication reduces the rate of complications; however, the benefits are undermined by rising rates of HP eradication treatment failure. This real-world observational cohort analysis aims to describe HP diagnostic and treatment patterns among insured patients in the US. Using diagnoses, lab results, and treatment patterns, we identified adults (18+) with new diagnoses of HP in the Veradigm Health Insights EHR Database linked to Komodo claims data (1/1/2016–12/31/2019). Patients were required to have ≥ 12 months of data pre-/post-index. We captured patient characteristics, HP-related diagnostic testing, and the use of US guideline-recommended HP eradication regimens. HP eradication rates following first-line eradication treatment were measured among patients with available lab results. Overall, 31.8% of the 60,593 included patients did not receive guideline-recommended treatment. Among the 68.2% (41,340) with first-line treatment, 80.2% received clarithromycin-based triple therapy, and 6.6% received bismuth quadruple therapy. Of the 4569 patients with a repeated course of eradication therapy, 53.4% received the same regimen as their first-line, the majority (90.7%) of whom received two rounds of clarithromycin-based triple therapy. Among the 2455 patients with results of HP non-serology testing following first-line treatment, the 180-day eradication rate was 80.2% overall, with differences based on treatments and demographics. This study highlights gaps between guideline-recommended HP management and real-world patterns, underscoring the need to improve HP testing, treatment, and follow-up practices.

## Introduction

*Helicobacter pylori* (HP) infects approximately 36% of the United States (US) population^1^, although there is variation in prevalence by birth cohort, race, ethnicity, socioeconomic status, and immigration status, among other factors^2–5^. Chronic HP infection invariably causes gastric inflammation; while most people are asymptomatic, chronic HP infection is associated with gastric and, less commonly, extragastric pathology. Chronic HP infection is causally associated with both peptic ulcer disease and gastric cancer (specifically, gastric adenocarcinoma and mucosa-associated lymphoid tissue lymphoma)^6^. HP is recognized by the WHO as a carcinogen and is the leading cause of infection-attributable cancer, responsible for over 80% of gastric cancer globally^7^. Worldwide, gastric cancer remains the fourth leading cause of cancer-related deaths^8^. In the US, gastric cancer is the third most common gastrointestinal cancer, but the incidence is markedly higher in certain groups, especially non-White populations^9^.

HP eradication is associated with a significantly lower risk of gastric cancer as well as peptic ulcer disease complications and recurrence compared to persistent infection^10–12^. Accordingly, current US guidelines recommend eradication therapy for all people diagnosed with active HP infection^13^. Bismuth quadruple therapy is the recommended and preferred first-line treatment, whereas clarithromycin-based triple therapy, is now a qualified first-line treatment, and should only be considered for individuals without prior macrolide exposure, those living in a region where HP resistance to clarithromycin is demonstrated to be less than 15%, and individuals with known clarithromycin-sensitive HP strains^14^. Clarithromycin-based triple therapy is no longer a preferred first-line treatment in the US due to steadily increasing resistance rates. If first-line treatment fails to eradicate HP, salvage regimens should avoid previously used antibiotics^15^.

It should be emphasized, though, that in the US, there are no large-scale HP registries to track local HP antibiotic resistance rates and regimen-specific success rates, nor are HP antibiotic susceptibility data easy to obtain. Indeed, the rates of successful eradication have been declining in the US, and increasing antibiotic resistance is one of the leading reasons for this. Based on limited data, the clarithromycin resistance rate increased from 9.1% in 2009–2010 to 24.2% in 2011–2013 among US Veterans^16^, and clarithromycin resistance is significantly associated with eradication failure^17^. Rising rates of HP eradication treatment failure undermine the potential benefits of HP eradication therapy and further contribute to increases in antibiotic resistance^18^. As such, current US guidelines recommend eradication confirmation testing with a non-serological HP test in all individuals, given that the only way to confirm successful HP eradication is via repeat non-serological testing, ideally at least 4 weeks post-treatment^13^.

Despite clear indicators of declining HP treatment success in the US, there are no US general population-level analyses describing real-world HP management patterns. Accordingly, the primary objective of this claims-based analysis is to describe HP diagnostic and treatment patterns in the US, focusing on the first two courses of HP treatment and highlighting differences based on patient demographics and relevant clinical factors.

## Methods

### Study design and data sources

We conducted a retrospective, observational cohort study using the Veradigm Health Insights Ambulatory EHR Research Database linked with insurance claims data from the Komodo Health Healthcare Map from January 1, 2015, to July 31, 2021 (hereafter, 'the linked dataset'). The Veradigm EHR Research Database is one of the largest datasets of US EHRs and consists of de-identified patient records sourced from ambulatory/outpatient primary care and specialty settings. The insurance claims data contain de-identified inpatient, outpatient, and pharmacy claims. Only closed claims were used for this study. These data are sourced from all 50 states and are representative of the insured population in the US.

Diagnoses were captured in claims and the EHR data using International Classification of Diseases (ICD), 9th Edition and 10th Edition, Clinical Modification (ICD-9-CM and ICD-10-CM) codes. Procedures were captured using ICD-9-PCS, ICD-10-PCS, Current Procedural Terminology (CPT), and Healthcare Common Procedure Coding System (HCPCS) codes. Lab test results were captured using Logical Observation Identifiers Names and Codes (LOINCs) and a non-structured field containing lab test names in the EHR.

### De-identification and ethical compliance

The linked dataset only contains de-identified data as per the de-identification standard defined in Section §164.514(a) of the Health Insurance Portability and Accountability Act of 1996 (HIPAA) Privacy Rule. The process by which the data is de-identified is attested to through a formal determination by a qualified expert as defined in Section §164.514(b)(1) of the HIPAA Privacy Rule. Because this study used only de-identified patient records, it is therefore no longer subject to the HIPAA Privacy Rule and is therefore exempt from Institutional Review Board approval and for obtaining informed consent according to US law. This study was conducted in compliance with the Declaration of Helsinki and used only de-identified data.

### Study cohort

**Figure 1:**
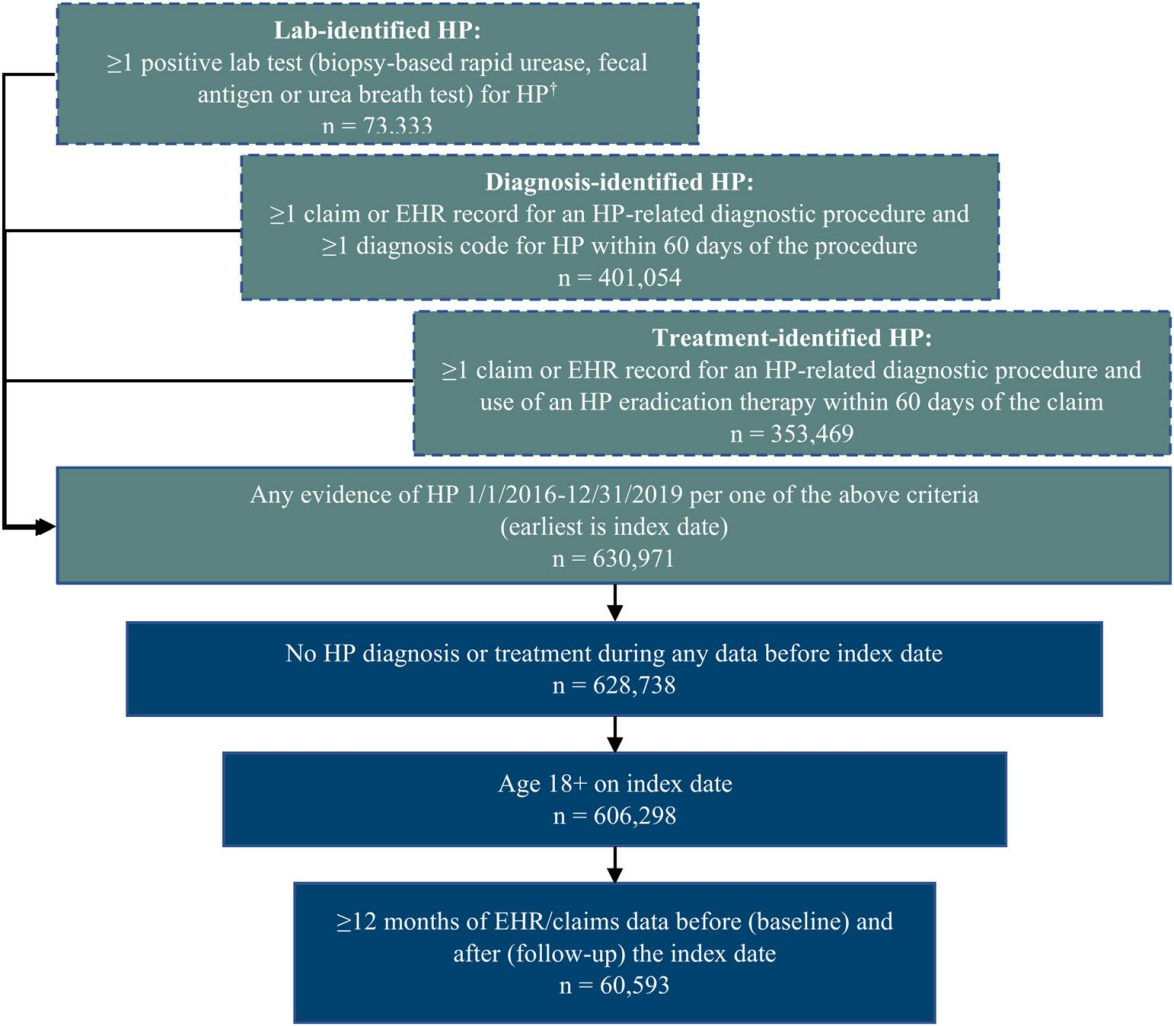
Patient selection criteria and resulting sample sizes. HP, *Helicobacter pylori*; EHR, electronic health record. ^†^Serology tests were not included as they are not used to confirm active HP infection.

Figure 1 illustrates the construction of the study cohort. We identified adult patients ≥ 18 years old in the linked dataset with HP infection based on one or more of the following criteria between January 1, 2016, and December 31, 2019: (1) HP based on positive laboratory test, (2) HP based on HP diagnostic test followed by a diagnosis code, (3) HP diagnostic test followed by the occurrence of HP eradication treatment. Of note, not all HP diagnostic tests documented in the claims database have associated results in the EHR database; as such, in order to preserve sample size while still reducing misclassification, we required that the test preceded the diagnosis code or HP treatment occurrence but did not require documentation of a test result for criteria #2 and #3. The earliest date on which patients met at least one of these three criteria was set as the index date.

Lab confirmation of HP was determined in a subset of patients with results for lab tests for active HP infection (i.e., rapid urease, fecal antigen, or urea breath tests [UBT]) available in the EHR (Supplementary Table S1). Diagnosis confirmation required the presence of at least one EHR encounter or medical claim with a diagnosis code for HP (ICD-9-CM: 041.86 or ICD-10-CM: B96.81), along with the additional requirement that the HP diagnosis be preceded by a claim or record for a diagnostic test for HP (esophagogastroduodenoscopy [EGD], rapid urease, fecal antigen, or UBT; Supplementary Table S1) in the 60 days before the diagnosis code. Finally, for HP treatment, we required that patients have pharmacy claims indicating they received an HP eradication regimen consistent with US guidelines (Supplementary Table S2)^13^. Patients were categorized as having received a specific HP regimen when prescription fills for all drugs in the regimen were observed within a 14-day period. Because proton-pump inhibitors (PPIs) are often purchased over-the-counter and, therefore, may not be captured in EHR or claims data, only the non-PPI medications in the eradication regimens were required to be present for a regimen to be identified. However, if a PPI was observed in the 30 days before through the 14 days after the start of a regimen, the PPI type and dose were recorded. To confirm that each regimen was being used to treat HP, we also required an HP-related diagnostic test (EGD, rapid urease, fecal antigen, or UBT) in the 60 days prior to each regimen.

To focus our analysis on patients with newly diagnosed HP, we excluded individuals with any evidence of lab-, diagnosis-, or treatment-identified HP during *any* available history in the linked dataset prior to the index date. We required continuous enrollment in the claims database and activity in the EHR for at least 12 months before (baseline period) and after (follow-up period) the index date. The follow-up period was variable in duration (minimum 12 months) and ended with the earliest of the end of continuous enrollment in the claims database, the end of activity in the EHR, or the end of available data in the linked dataset.

### Study variables

Demographic characteristics were recorded at the index date. We also recorded body mass index (BMI), HP-related diagnoses, and medications from the EHR and claims (pharmacy claims only for medications) during the 12 months prior to the index date, hereafter referred to as the "baseline period".

All US guideline-recommended HP eradication regimens (Supplementary Table S2) that occurred during the individual follow-up period were recorded from pharmacy claims per the methodology described above. Prescriptions for *any* antibiotic medications or PPIs were also recorded during the baseline and follow-up periods, separate from those recorded as part of HP eradication treatment.

To evaluate testing patterns, we recorded any occurrences of EGD procedures, rapid urease tests, fecal antigen tests, UBT, and serum antibody tests in the baseline period and following first-line eradication treatment (Supplementary Table S1).

### Eradication rate analysis

Among patients with at least one eradication regimen during follow-up, we identified the subgroup with at least 180 days of enrollment in claims and activity in the EHR following the end of first-line therapy. In this group, we identified those with available HP lab test results within 180 days after the end of first-line therapy. The post-treatment eradication rate was calculated as the number of patients with negative HP lab test results divided by the total number of patients with any HP lab test results.

### Data analysis

This analysis was descriptive in nature. Descriptive statistics were generated using SAS V9.4.

## Results

### Cohort characteristics

After applying the study selection criteria, the final analytic cohort consisted of 60,593 patients with HP infection between 2016 and 2019 (Fig. 1). Cohort characteristics are provided in Table 1. The mean age was 54.2 (SD: 14.5) years, and 65.5% were female. Nearly 70% were overweight or obese based on index BMI. The mean (SD) follow-up was 27.5 (17.4) months (median: 25.7 months, IQR: 23.7).

**Table 1:** Characteristics of patients with newly diagnosed Helicobacter pylori infection during the 12-month baseline period.

	All patients
	(n = 60,593)
Age at index, mean (SD)	54.2 (14.5)
Female, N (%)	39,699 (65.5)
BMI	29.9 (7.0)
< 18.5 (underweight)	834 (1.4)
18.5–24.9 (healthy weight)	11,970 (19.8)
25.0–29.9 (overweight)	17,950 (29.6)
30–39.9 (obese)	18,981 (31.3)
≥ 40 (morbidly obese)	5405 (8.9)
Unknown/not reported	5453 (9.0)
Clinical conditions, N (%)	
Cardiovascular disease	37,814 (62.4)
Hypertension (primary)	33,159 (54.7)
Diabetes	19,578 (32.3)
*H. pylori*-related conditions^a^, N (%)	
Abdominal pain/tenderness	35,929 (59.3)
GERD	29,803 (49.2)
Gastritis	23,885 (39.4)
Dyspepsia	18,497 (30.5)
Esophagitis	9326 (15.4)
Gastric/duodenal ulcer	4132 (6.8)
Gastric polyp	1518 (2.5)
Gastric cancer	147 (0.2)
Medications, N (%)	
Any antibiotic	33,398 (55.1)
Penicillins (e.g., amoxicillin)	16,409 (27.1)
Macrolides (e.g., clarithromycin)	13,251 (21.9)
Fluoroquinolones (e.g., levofloxacin)	10,565 (17.4)
Proton pump inhibitors (PPIs)	28,516 (47.1)
NSAIDs/COX-2 inhibitors	24,590 (40.6)
Corticosteroids (oral)	13,068 (21.6)
H2-receptor antagonists	10,579 (17.5)

COX, cyclooxygenase-2; GERD, gastroesophageal reflux disease; H2, histamine type 2; NSAID, Non-steroidal anti-inflammatory drug.

^a^Presence of conditions determined by diagnosis codes and did not require having a prior claim for diagnostic testing such as endoscopy.

The most common general comorbidities were cardiovascular disease (62.4%), primary hypertension (54.7%), and diabetes (32.3%). Prevalent gastroenterological conditions and symptoms during the 12-month baseline period included abdominal pain/tenderness (59.3%), gastroesophageal reflux disease (49.2%), gastritis (39.4%), and dyspepsia (30.5%) (Table 2). Peptic ulcers were reported in approximately 6.8%, and 0.2% of the cohort had diagnostic codes for peptic ulcer disease or gastric cancer, respectively, during the baseline period. In the 12 months prior to HP diagnosis, 55.1% of the cohort filled at least one prescription for an antibiotic: 21.9% received a macrolide antibiotic and 17.4% a fluoroquinolone during this period. Nearly half (47.1%) of the cohort filled a prescription for a PPI during the baseline period.

**Table 2:** *Helicobacter pylori* (HP) eradication regimens.

	HP eradication regimen 1 (n = 41,340)	HP eradication regimen 2 (n = 4569)	HP eradication regimen 3 (n = 972)
Eradication therapy, n (%)			
Clarithromycin triple	33,142 (80.2%)	2499 (54.7%)	439 (45.2%)
Clarithromycin triple fixed-dose combination	4628 (14.0%)	243 (9.7%)	36 (8.2%)
Bismuth quadruple	2748 (6.6%)	809 (17.7%)	164 (16.9%)
Bismuth quadruple fixed-dose combination	2233 (81.3%)	541 (66.9%)	113 (68.9%)
Concomitant/sequential/hybrid	2122 (5.1%)	264 (5.8%)	75 (7.7%)
Levofloxacin triple	1912 (4.6%)	600 (13.1%)	153 (15.7%)
High-dose dual	1067 (2.6%)	160 (3.5%)	49 (5.0%)
Levofloxacin sequential	213 (0.5%)	77 (1.7%)	18 (1.9%)
LOAD	84 (0.2%)	53 (1.2%)	16 (1.6%)
Rifabutin triple	52 (0.1%)	107 (2.3%)	58 (6.0%)

LOAD, levofloxacin, omeprazole, Alinia [nitazoxanide], and doxycycline.

### HP eradication therapies

Of 60,593 patients with newly diagnosed HP infection, 68.2% (n = 41,340) received at least one US guideline-recommended HP eradication therapy during the follow-up period (hereafter, ‘guideline-treated patients’) (Table 2). The majority (88.9%, n = 36,771) of guideline-treated patients received one course of guideline-recommended treatment during follow-up, while 11.1% (n = 4569) had two or more treatment courses, and 2.4% (n = 972) had three or more treatment courses during the observed follow-up period (Table 2). Among the 31.8% (n = 19,253) who did not receive a guideline-recommended treatment, 89.3% received an antibiotic, and 83.2% filled a PPI prescription at least one time during follow-up.

During the observation period (2016–2021), clarithromycin triple therapy was the most commonly used first-line HP treatment and was prescribed to 80.2% of guideline-treated patients. Bismuth quadruple therapy (6.6%), concomitant/sequential/hybrid therapy (5.1%), levofloxacin triple therapy (4.6%), and high-dose dual therapy (2.6%) were used infrequently as first-line treatment. LOAD, levofloxacin sequential, and rifabutin triple therapies together accounted for < 1% of first-line therapies. Across all therapy types, the mean duration of first-line treatment was 14.2 days (SD: 4.7 days; median:14 days). The majority of guideline-treated patients (73.6%) filled a PPI prescription as part of first-line treatment using their insurance; 65.1% were prescribed omeprazole, 23.8% lansoprazole, 15.8% pantoprazole, and the remaining 3.1% used an alternative PPI. First- and second-line therapies, stratified by the calendar year in which the treatment was initiated, are provided in Supplementary Table S3. This predominance of clarithromycin triple therapy persisted even after the publication of updated ACG guidelines in 2017, with 78.1% of those who initiated treatment in 2019 receiving clarithromycin triple therapy.

Among the 85.3% (n = 35,264) of patients who received a clarithromycin-containing regimen as first-line therapy (clarithromycin triple therapy or concomitant/sequential/hybrid therapy), 21.0% had a prescription for a macrolide antibiotic recorded during the 12-month baseline period. While only 5.3% of guideline-treated patients used a levofloxacin-based regimen (levofloxacin triple or sequential therapy or LOAD) as first-line therapy, 35.3% had a prescription for a fluoroquinolone antibiotic during baseline. Overall, 45.3% (n = 27,445) of patients either did not receive a guideline-recommended treatment or received a potentially inappropriate regimen based on prior antibiotic use.

Among the 11.1% (n = 4569) of guideline-treated patients who received a second US guideline-recommended eradication regimen, clarithromycin triple therapy remained the most common (54.7%) regimen for second-line treatment, followed by bismuth quadruple therapy (17.7%) and levofloxacin triple therapy (13.1%). The mean duration of second-line treatment was 14.3 days (SD: 7.4 days; median: 14 days). Similar to first-line, 70.4% of guideline-treated patients filled a PPI prescription as part of second-line treatment; 69.2% were prescribed omeprazole, 16.9% lansoprazole, 17.9% pantoprazole, and the remaining 3.9% used an alternative PPI.

**Figure 2:**
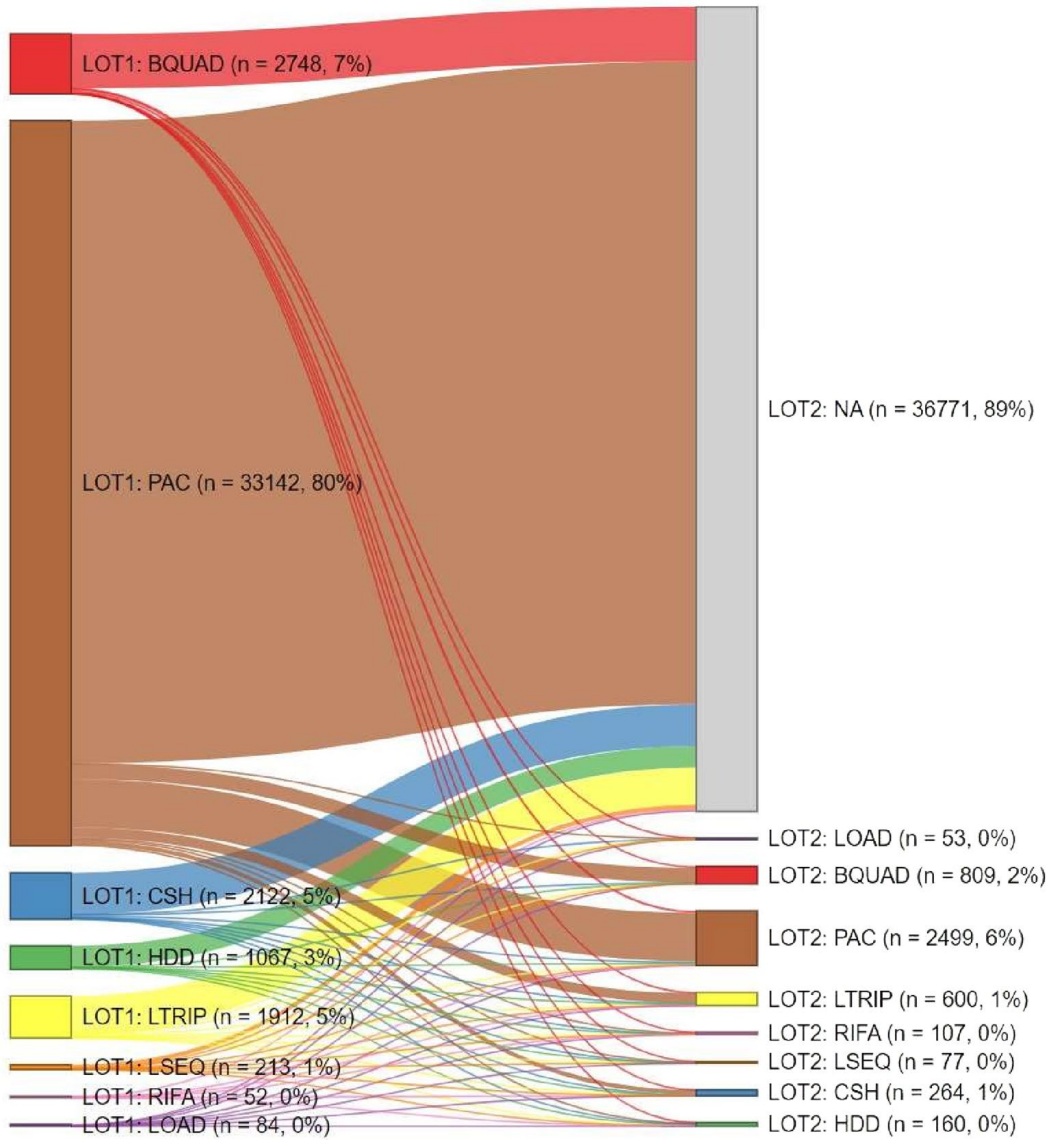
Transitions from first-line to second-line *Helicobacter pylori* eradication therapies. BQUAD, bismuth quadruple therapy; CSH, concomitant, sequential, or hybrid therapy; HDD, high-dose dual therapy (amoxicillin, PPI); LOAD, levofloxacin/nitazoxanide/doxycycline/PPI therapy; LOT, line of therapy; LSEQ, levofloxacin sequential therapy; LTRIP, levofloxacin triple therapy; PAC, PPI, amoxicillin, clarithromycin; PPI, proton pump inhibitor; RIFA, rifabutin triple therapy.

Transitions from first- to second-line eradication therapy are illustrated in Fig. 2. Among the 11.1% with at least one additional course of HP treatment, 53.5% (n = 2443) received the same treatment regimen for first- and second-line (e.g., clarithromycin triple therapy for both first- and second-line treatment). The most common first- to second-line treatment sequences were clarithromycin triple therapy to clarithromycin triple therapy (48.5%), clarithromycin triple therapy to bismuth quadruple therapy (14.8%), and clarithromycin triple therapy to levofloxacin triple therapy (10.1%). The median (IQR) time between the end of the first treatment and the start of the second treatment was 153 (414) days.

### HP testing patterns and eradication rates

Diagnostic testing patterns in the 60 days prior to and including the index date are provided in Supplementary Table S4. As a function of the patient selection criteria, every patient had at least one diagnostic test for HP. In the 60 days prior to HP diagnosis, 65.5% of patients had a diagnostic EGD procedure, 25.5% had a UBT, 9.4% had a fecal antigen test: 4.2% had a serum antibody test, and 0.1% had a rapid urease test.

US guidelines recommend confirmation of HP eradication testing with a non-serological HP test at least 4 weeks after completion of antibiotic therapy. Of patients who received guideline-recommended eradication treatment, 32.6% had no follow-up HP diagnostic tests to confirm eradication (Supplementary Table S5). Among the 67.4% of patients who did receive at least one follow-up test, 46.2% had a UBT (median [IQR]: 75 [254] days), 28.3% had a diagnostic EGD procedure (median [IQR]: 235 [588] days), and 23% had a fecal antigen test as their first test (median [IQR]: 64 [144] days). A small number (< 3%) had a serum antibody test as their first post-eradication treatment test. Supplementary Table S5 shows all diagnostic tests received by guideline-treated patients after first-line treatment.

To estimate the HP eradication rate following first-line therapy, we first identified a subset of patients with at least 180 days of available data after first-line treatment (n = 41,173, or 99.6% of guideline-treated patients); among this group, 6.0% (n = 2455) had non-serological HP lab test *results* available within 180 days of the end of first-line treatment. Among this subgroup, 79.8% (n = 1960) had a negative post-eradication treatment HP test result, while 20.2% had a persistent positive non-serological HP test after first-line therapy. The 180-day eradication rate was 80.2% for the 2125 patients treated with clarithromycin triple therapy (N = 1215), 85.5% for the 124 patients treated with bismuth quadruple therapy, and 78.9% for the 123 patients treated with concomitant/sequential/hybrid therapy.

Table 3 shows 180-day eradication rates following first-line therapy stratified by patient characteristics (e.g., age, sex). The eradication rate was 83.5% among males and 78.1% among females. Among patients with a reported race, the eradication rate was 86.6% for White individuals, 80.5% for Black individuals, and 82.5% for Asian individuals. Eradication rates varied geographically, with the Northeast having the highest rate (80.3%) and the Midwest with the lowest rate (74.9%).

**Table 3:** *Helicobacter pylori* (HP) eradication rates after first-line (1L) eradication treatment.

	With 1L therapy + 90D follow-up, n	With 1L therapy + 180D follow-up, n	Lab-confirmed eradication rate, 90 days		Lab-confirmed eradication rate, 180 days	
			Any HP lab result, n	Negative lab result, n (%)	Any HP lab result, n	Negative lab result, n (%)
Overall	41,216	41,173	1906	1469 (77.1)	2455	1960 (79.8)
Age group						
18–64	31,168	31,135	1376	1058 (76.9)	1794	1421 (79.2)
≥ 65	9165	9158	499	388 (77.8)	617	501 (81.2)
Unknown/not reported	883	880	31	23 (74.2)	44	38 (86.4)
Gender						
Male	13,982	13,977	621	501 (80.7)	787	657 (83.5)
Female	27,227	27,189	1284	967 (75.3)	1667	1302 (78.1)
Unknown/not reported	7	7	1	1 (100.0)	1	1 (100.0)
Race						
White	4462	4457	295	248 (84.1)	351	304 (86.6)
Black	1295	1290	77	60 (77.9)	113	91 (80.5)
Asian	376	376	47	37 (78.7)	57	47 (82.5)
Other	509	508	47	40 (85.1)	56	46 (82.1)
Unknown/not reported	34,574	34,542	1440	1084 (75.3)	1878	1472 (78.4)
BMI						
< 18.5 (underweight)	562	562	32	19 (59.4)	40	27 (67.5)
18.5–24.9 (healthy weight)	8126	8117	494	382 (77.3)	630	504 (80.0)
25.0–29.9 (overweight)	12,119	12,107	667	521 (78.1)	857	695 (81.1)
30–39.9 (obese)	12,933	12,919	554	421 (76.0)	731	571 (78.1)
≥ 40 (morbidly obese)	3749	3745	127	105 (82.7)	153	126 (82.4)
Unknown/not reported	3727	3723	32	21 (65.6)	44	37 (84.1)
Geographic region						
Northeast	9634	9631	305	245 (80.3)	404	332 (82.2)
Midwest	4552	4549	183	137 (74.9)	234	175 (74.8)
South	13,929	13,907	752	579 (77.0)	950	761 (80.1)
West	9032	9021	534	407 (76.2)	693	554 (79.9)
Other/unknown	4069	4065	132	101 (76.5)	174	138 (79.3)

BMI, body mass index; D, days.

## Discussion

This is the first US population-based analysis to review current HP treatment patterns and eradication rates in the US based on real-world data. In this analysis of US claims and EHR data, 45.3% of patients with HP infection either did not receive a guideline-recommended treatment or received a potentially inappropriate regimen based on prior antibiotic use. Clarithromycin triple therapy was the most commonly prescribed first- and second-line regimen despite macrolide exposure in the baseline period for over 20% of patients, and first-line clarithromycin triple therapy use was observed in nearly half of patients who received second-line clarithromycin triple therapy. Roughly one-third of patients did not receive post-treatment HP eradication testing, and among the sub-group of patients with available data, 20% had HP eradication treatment failure with first-line therapy. These real-world findings suggest there are opportunities to significantly improve HP treatment and management gaps in line with current evidence-based guidelines.

The most recent ACG guidelines, published in 2017, recommend against using clarithromycin triple therapy as first-line *unless* specific criteria are met: (1) regional clarithromycin resistance rates are lower than 15%, (2) the patient does not have precious macrolide exposure, and (3) the specific strain has been shown to be clarithromycin-sensitive^13^. In the present study, over 80% of patients received clarithromycin triple therapy as first-line, and over one-half received it as second-line, which is consistent with previous smaller studies but the first to demonstrate it in a general US population^19–21^. Our findings suggest the first-line regimen utilized was often inappropriate based on patients' prior antibiotic exposure. For example, 21.0% of patients who received a clarithromycin-containing first-line regimen had a prescription for a macrolide antibiotic during just the 12-month pre-index period alone. Notably, as HP is typically acquired during childhood^5^, the percentage of patients who received a macrolide prescription following infection but preceding first-line HP eradication therapy is likely even higher. Our findings are consistent with a previous analysis of the PharMetrics Plus database, which found that 18% of patients who received a clarithromycin-based first-line regimen had received a macrolide prescription in the preceding year^19^. We observed similar trends with first-line levofloxacin-containing regimens and prior fluoroquinolone exposure.

While local resistance rates are not widely available, recent US studies have consistently found resistance to clarithromycin to be greater than 15%^16,17,22,23^. Despite this, our analysis showed that clarithromycin prescribing patterns remained fairly steady during the study period decreasing only slightly from 81.4% in 2016 to 78.1% in 2019. Of note, the majority of our study timeframe preceded the approval of fixed-dose rifabutin triple therapy and would not capture the impact of this approval on treatment patterns. Despite being strongly recommended for use as a first-line therapy, only 6.6% of patients received bismuth quadruple therapy in the first-line setting. Furthermore, the choice of second-line therapy was often inappropriate as approximately one-half (50%) of patients who received second-line therapy received the same treatment as during the first-line, contrary to recommended guidelines. Contrary to guideline recommendations, over 90% of patients who received the same first- and second-line therapy received two cycles of clarithromycin triple therapy^15^.

The recently updated Maastricht V/Florence Consensus Report suggested susceptibility testing prior to prescribing first-line treatment to align with antibiotic stewardship practices; despite the recommendation, they note that routine utilization of susceptibility testing in clinical practice remains to be established and may not always be feasible^14^. In the absence of susceptibility testing, the treating physician should utilize local resistance patterns of antibiotics to guide decision-making around the use of clarithromycin triple therapy as a first-line treatment^13,14^. Herein remains a knowledge gap due to the lack of a US national registry with local and regional resistance patterns, which may explain the continued heavy reliance on clarithromycin triple therapy.

In addition to gaps between recommended eradication treatment and real-world treatment patterns, this study also identified gaps between recommended and real-world post-eradication treatment testing practices. As previously noted, persistent HP infection places patients at ongoing risk of complications related to chronic HP infection. In the face of rising antibiotic resistance rates and rising eradication failure rates, it is critical that patients undergo repeat non-serological testing, ideally at least 4 weeks following the completion of eradication treatment, in order to confirm eradication. Furthermore, resolution (and likewise, persistence) of symptoms does not correlate with the success versus failure of eradication treatment and thus is not a replacement for non-serology post eradication treatment testing. In this claims-based study, only 67.4% of patients received a diagnostic HP test after first-line therapy to confirm successful eradication. While this is higher than previously reported rates, which ranged from 24 to 44%^24,25^, it still suggests that one-third of HP patients complete eradication therapy without evaluating if that eradication was successful. Further investigation is needed into whether this is due to a missed opportunity of healthcare providers to recommend testing or a missed opportunity of patients to return for physician-recommended testing.

Indeed, in the subset of patients with results of post-treatment non-serologic HP testing available, over 20% demonstrated persistent HP infection following first-line therapy, including 20% of patients who received clarithromycin triple therapy and 15% of those who received bismuth quadruple therapy. These findings are consistent with other real-world US studies, including one of 1966 US military personnel that reported eradication failure in 25% of all patients, 24% failure among patients who received clarithromycin triple therapy, and 15% failure among patients who received bismuth quadruple therapy^26^. Similarly, a retrospective analysis of 1101 patients treated in Rhode Island reported eradication failure in 23% of patients who received clarithromycin triple therapy and 14% of patients who received bismuth quadruple therapy^27^.

Our data suggest that not all patients who experienced HP eradication treatment failure went on to receive subsequent treatment. Given the limited number of post-eradication treatment confirmation testing, we were limited in our ability to discriminate the eradication failure rate for the entire cohort. That said, only 11% of patients who received first-line therapy went on to receive second-line therapy. As an 89% overall eradication rate is unlikely, given the findings of the subset analysis and prior studies, this suggests many patients may have persistent HP infection. Potential reasons for this discrepancy include that some providers may not offer treatment if patients report symptom resolution or that patients who experience resolution or significant improvement of symptoms may be less inclined to complete another course, particularly considering that side effects are common with HP treatment. As noted above, resolution of symptoms does not equate to HP eradication, and patients remain at risk for downstream complications in the face of persistent HP infection.

### Limitations

This retrospective analysis used routinely collected claims and EHR data and is subject to the typical limitations of using retrospective data. The dataset used in this analysis included insured individuals, which may additionally impact generalizability to the entire US population, as roughly 15% of US adults aged 18 to 64 were uninsured in 2019^28^. These findings also may not be generalizable to individuals with certain types of insurance not captured in this dataset, such as those who receive their healthcare through the Veterans Health Administration. Additionally, only a small subset of patients had non-serological lab results available following HP eradication treatment, which may impact the accuracy of our estimated eradication rate, as well as generalizability since patients who had repeat testing ordered and completed may represent a higher risk group compared to the group of patients who did not.

We used a strict definition of guideline-recommended regimens, so we may have missed patients who received alternative regimens outside of guideline recommendations but still with the intention of treating HP. We are not able to discriminate between concomitant, sequential, and hybrid therapy using claims data, and we, therefore, combined these regimens; while we cannot make separate conclusions for these treatments, they comprised a minority of regimens: 5.1% of first-line and 5.8% of second-line regimens. As with any retrospective data analysis, we cannot confirm patient adherence to treatment even if the full prescription course is filled.

We were not able to determine the exact date of HP acquisition, which is a common limitation that is difficult to overcome. Previous studies in endemic populations suggest HP acquisition most often occurs in early childhood, which is supported by limited US data, at least among families with an infected parent^29^. In addition, we are unable to confirm details regarding the diagnostic EGD, including whether or not gastric biopsies were obtained and, if so, the number and location; whether intraprocedure HP tests were performed; nor can we confirm whether special pathology stains were used for HP detection on gastric biopsies. Similarly, the use of HP antibody sensitivity testing was very rare, and we cannot comment on the antibody sensitivity of HP strains.

Finally, our follow-up time, while sufficient to determine treatment and post-treatment practice patterns, is insufficient to thoroughly evaluate the downstream consequences of eradication failure. This is an important area of research in light of rising eradication failure rates. Future research should explore whether the implementation of order sets or other clinical decision support tools can increase guideline adherence and improve patient outcomes.

## Conclusions

Persistent HP infection has potentially serious downstream consequences. This study highlights that there are still gaps in the management of HP in terms of treatment selection for both first and second-line therapy. Likewise, there are major opportunities to improve post-eradication testing and registration of patients undergoing treatment in order to track regimen-specific eradication success rates locoregionally across the US.

## Supplementary Information

Supplementary Tables.

## Data Availability

The data that support the findings of this study were used under license from Veradigm and Komodo Health. Due to data use agreements and its proprietary nature**,** restrictions apply regarding the availability of the data. Further information is available from the corresponding author.
