# Supplementary material for: Diagnosis and treatment patterns among patients with newly diagnosed Helicobacter pylori infection in the United States 2016–2019
Source: Sci Rep. 2023 Jan 25;13:1375. doi: 10.1038/s41598-023-28200-3 (PMC9876904; doi:10.1038/s41598-023-28200-3)
Supplement: Supplementary file 1 — Supplementary Tables. [file 41598_2023_28200_MOESM1_ESM.pdf]

Supplementary Information for: **Diagnosis and Treatment Patterns Among Patients with Newly Diagnosed *Helicobacter pylori* Infection in the United States 2016-2019: A Linked Electronic Medical Record-Claims Database Analysis**

Table S1. Code Sets used to Identify *Helicobacter pylori* (HP) Labs and Procedures

Table S2. American College of Gastroenterology Recommended *Helicobacter pylor*i Eradication Regimens

Table S3. First- and Second-Line Therapies by Calendar Year

Table S4. Diagnostic Testing During the Baseline Period (including index date)

Table S5. Diagnostic Test After Completion of First-Line Eradication Treatment

Table S1. Code Sets used to Identify *Helicobacter pylori* (HP) Labs and Procedures

Description	Code Type	Code
Urea breath test	LOINC	29891-9, 29892-7
	CPT	78267, 78268, 83013, 83014
Esophagogastroduodenoscopy (diagnostic; used in patient selection)	CPT	43235, 43237, 43238, 43239, 43242, 43252, 43253, 43259, 0652T, 0653T, 0654T
Esophagogastroduodenoscopy (any)	CPT	43210, 43233, 43235, 43236, 43237, 43238, 43239, 43240, 43241, 43242, 43243, 43244, 43245, 43246, 43247, 43248, 43249, 43250, 43251, 43252, 43253, 43254, 43255, 43256, 43257, 43258, 43259, 43266, 43270, 0652T, 0653T, 0654T
Fecal antigen	LOINC	87952-8, 31843-6, 17780-8, 80373-4
	CPT	87338
Serum antibody	LOINC	51762-3, 22310-7, 6419-6, 7900-4, 16533-2, 5174-8, 22311-5, 16929-2, 5175-5, 16125-7, 40820-3, 7901-2, 6420-4, 16126-5, 60524-6, 17859-0, 26984-5, 7902-0, 5176-3, 16127-3, 46987-4, 7903-8, 40821-1, 5177-1
	CPT	86677
Unknown HP test type	LOINC	45037-9, 44015-6, 587-6, 49101-9, 91060-4, 91061-2
	CPT	87339, 83009
Rapid urease test	LOINC	32637-1, 87953-6, 50320-1, 42640-3, 43805-1, 43806-9, 43855-6, 66491-2, 43808-5, 42636-1, 66492-0, 43807-7

CPT, Current Procedural Terminology; LOINC, Logical Observation Identifiers Names and Codes

Table S2. American College of Gastroenterology Recommended *Helicobacter pylor*i Eradication Regimens

**Eradication Regimen**	**Drug 1†**	**Drug 2**	**Drug 3**	**Drug 4**
Clarithromycin triple	PPI	clarithromycin	amoxicillin	n/a
		clarithromycin	metronidazole	n/a
Bismuth quadruple	PPI	bismuth	tetracycline	metronidazole
Concomitant/Sequential/Hybrid	PPI	clarithromycin	amoxicillin	metronidazole
		clarithromycin	amoxicillin	tinidazole
Levofloxacin triple	PPI	levofloxacin	amoxicillin	n/a
Levofloxacin sequential	PPI	levofloxacin	amoxicillin	metronidazole
		levofloxacin	amoxicillin	tinidazole
LOAD	PPI	levofloxacin	nitazoxanide	doxycycline
Rifabutin Triple	PPI	amoxicillin	rifabutin	n/a
High-dose dual	PPI	amoxicillin (>3000mg/day)	n/a	n/a

n/a, not applicable; PPI, proton pump inhibitor.

†PPI use was captured but not required for regime confirmation due to high expected use of over-the-counter PPIs.

Table S3. First- and Second-Line Therapies by Calendar Year

	2016	2017	2018	2019
**First-Line HP Eradication Therapy**				
Total, n	11473	11828	10258	7781
Clarithromycin Triple, n (%)	9,339 (81.4)	9,515 (80.4)	8,211 (80.0)	6,077 (78.1)
Bismuth Quadruple, n (%)	843 (7.3)	783 (6.6)	606 (5.9)	516 (6.6)
Levofloxacin Triple, n (%)	457 (4.0)	493 (4.2)	531 (5.2)	431 (5.5)
Concomitant/Sequential/Hybrid, n (%)	445 (3.9)	615 (5.2)	569 (5.5)	493 (6.3)
High-dose Dual, n (%)	310 (2.7)	308 (2.6)	245 (2.4)	204 (2.6)
Levofloxacin Sequential, n (%)	51 (0.4)	74 (0.6)	59 (0.6)	29 (0.4)
LOAD, n (%)	23 (0.2)	23 (0.2)	22 (0.2)	16 (0.2)
Rifabutin Triple†, n (%)	5 (0.0)	17 (0.1)	15 (0.1)	15 (0.2)
**Second-Line HP Eradication Therapy**				
Total, n	577	950	1165	1163
Clarithromycin Triple, n (%)	304 (52.7)	503 (52.9)	634 (54.4)	641 (55.1)
Bismuth Quadruple, n (%)	151 (26.2)	194 (20.4)	207 (17.8)	183 (15.7)
Levofloxacin Triple, n (%)	66 (11.4)	117 (12.3)	167 (14.3)	157 (13.5)
Concomitant/Sequential/Hybrid, n (%)	18 (3.1)	59 (6.2)	66 (5.7)	79 (6.8)
High-dose Dual, n (%)	16 (2.8)	37 (3.9)	30 (2.6)	49 (4.2)
Levofloxacin Sequential, n (%)	8 (1.4)	13 (1.4)	28 (2.4)	21 (1.8)
Rifabutin Triple†, n (%)	8 (1.4)	13 (1.4)	16 (1.4)	20 (1.7)
LOAD, n (%)	6 (1.0)	14 (1.5)	17 (1.5)	13 (1.1)

†Talicia (a fixed-dose combination medication containing rifabutin, omeprazole, and amoxicillin) was approved by the FDA in November 2019

Note; This analysis is at the line of therapy level, not the patient level (ie. N's represent lines of therapy, not patients)

Table S4. Diagnostic Testing During the Baseline Period (including index date)

	**All Patients (n = 60,593)**
≥1 diagnostic test, N (%)	60,593 (100)
Biopsy-based rapid urease test	78 (0.1)
Esophagogastroduodenoscopy	39,713 (65.5)
Fecal antigen	5,712 (9.4)
Serum antibody	2,542 (4.2)
Urea breath test	15,435 (25.5)

SD, standard deviation.

Table S5. Diagnostic Test After Completion of First-Line Eradication Treatment

	**All Patients with 1L Eradication Treatment (n = 41,340)**		
	**Patients, n (%)**	**Days to Test, mean (SD)**	**Days to Test, median (IQR)**
**First diagnostic test**			
No lab test	13,462 (32.6)		
First lab test	27,878 (67.4)	227.9 (326.6)	75 (254)
Urea breath test	12,885 (46.2)	149.7 (236.0)	56 (108)
Esophagogastroduodenoscopy	7,882 (28.3)	399.5 (425.8)	235 (588)
Fecal antigen	6,424 (23.0)	161.4 (243.7)	64 (144)
Serum antibody	630 (2.3)	353.5 (372.1)	225.5 (454)
Unknown test type	36 (0.1)	305.9 (451.2)	120 (372)
Rapid urease test	21 (0.1)	206.4 (373.7)	34 (67)
**All diagnostic tests**†			
Urea breath test	15,018 (53.9)	202.0 (301.1)	68 (196)
Esophagogastroduodenoscopy	14,130 (50.7)	490.1 (441.4)	368 (654)
Fecal antigen	8,297 (29.8)	249.3 (339.9)	91 (297)
Serum antibody	1,225 (3.0)	478.2 (425.2)	356 (588)
Unknown test type	188 (0.7)	419.9 (457.5)	229.5 (578)
Rapid urease test	38 (0.1)	303.4 (363.6)	125.5 (454)

SD, standard deviation.

† Patients may appear in more than one row below (e.g. if a patient had both EGD and an antibody test the patient would appear in both rows).
